# Factors associated with mortality of pediatric sepsis patients at the pediatric intensive care unit in a low-resource setting

**DOI:** 10.1186/s12887-021-02945-0

**Published:** 2021-10-25

**Authors:** Desy Rusmawatiningtyas, Arini Rahmawati, Firdian Makrufardi, Nurul Mardhiah, Indah Kartika Murni, Cuno S. P. M. Uiterwaal, Ary I. Savitri, Intan Fatah Kumara, Nurnaningsih

**Affiliations:** 1 Department of Child Health, Faculty of Medicine, Public Health and Nursing, Universitas Gadjah Mada/Dr. Sardjito Hospital, Yogyakarta, 55281 Indonesia; 2 Clinical Epidemiology, Julius Center for Health Sciences and Primary Care, University Medical Center, Utrecht, the Netherlands

**Keywords:** Sepsis, Mortality, Pediatric intensive care unit, Low-resource setting, Congenital anomaly, Fluid overload, Mechanical ventilation, Vasoactive drug

## Abstract

**Background:**

Sepsis is the leading cause of death worldwide in pediatric populations. Studies in low-resource settings showed that the majority of pediatric patients with sepsis still have a high mortality rate.

**Methods:**

We retrospectively collected records from 2014 to 2019 of patients who had been diagnosed with sepsis and admitted to PICU in our tertiary hospital. Cox proportional hazard regression modeling was used to evaluate associations between patient characteristics and mortality.

**Results:**

Overall, 665 patients were enrolled in this study, with 364 (54.7%) boys and 301 (46.3%) girls. As many as 385 patients (57.9%) died during the study period. The median age of patients admitted to PICU were 1.8 years old with interquartile range (IQR) ±8.36 years and the median length of stay was 144 h (1–1896 h). More than half 391 patients (58.8%) had a good nutritional status. Higher risk of mortality in PICU was associated fluid overload percentage of > 10% (HR 9.6, 95% CI: 7.4–12.6), the need of mechanical ventilation support (HR 2.7, 95% CI: 1.6–4.6), vasoactive drugs (HR 1.5, 95% CI: 1.2–2.0) and the presence of congenital anomaly (HR 1.4, 95% CI: 1.0–1.9). On the contrary, cerebral palsy (HR 0.3, 95% CI: 0.1–0.5) and post-operative patients (HR 0.4, 95% CI: 0.3–0.6) had lower mortality.

**Conclusion:**

PICU mortality in pediatric patients with sepsis is associated with fluid overload percentage of > 10%, the need for mechanical ventilation support, the need of vasoactive drugs, and the presence of congenital anomaly. In septic patients in PICU, those with cerebral palsy and admitted for post-operative care had better survival.

## Background

Sepsis is the leading cause of death worldwide in the pediatric population resulting in an estimated 7.5 million deaths annually [1]. Sepsis in pediatrics is generally defined as systemic inflammatory response syndrome (SIRS) with a suspected source of infection, whereas SIRS is described as a nonspecific inflammatory process occurring after trauma, infection, burns, pancreatitis, and other diseases [2, 3].

A study in a low-resource setting showed that 58% of pediatric patients with sepsis died, 58% had inotropic support, 18% had bloodstream infection, and 58% were supported with mechanical ventilation [4]. While pediatric sepsis mortality in high-resource settings was lower (with a rate of 5 to 20%) compared to low-resource settings [5], age was not found to be significantly different between both settings [6].

The high number of fatalities in low-resource settings caused by sepsis and its severity led us to study patient characteristics that were associated with mortality due to sepsis. A study showed that age, gender, duration of symptoms and laboratory results such as hemoglobin level, total leukocyte count, platelet count, pH, lactate and lactate clearance were associated with mortality in children with sepsis [7]. Another study investigated more characteristics with the addition of diagnosis at PICU transfer, anthropometry, and for laboratory parameters they used differential leukocyte count, serum C-reactive protein, blood urea, serum creatinine, prothrombin time and blood gas analysis results [4]. At the time of discharge/death, they recorded the following data: duration of PICU stay, duration of mechanical ventilation, number, and duration of inotropes required. The Pediatric Risk of Mortality III (PRISM III) scoring and number of organ dysfunction were obtained from all enrolled patients as well, but no association was found [4, 7]. In low-resource settings, studies on outcomes of children with sepsis treated in the Pediatric Intensive Care Unit (PICU) are still limited while findings from studies in other settings may not be applicable. Moreover, many previous studies in low-resource settings only provided the number of pediatric mortality but did not look for the factors that caused it [8]. This study aimed to evaluate the outcome of patients with sepsis admitted to PICU and its associated factors in a low-resource setting.

## Methods

### Study design and population

**Fig. 1:**
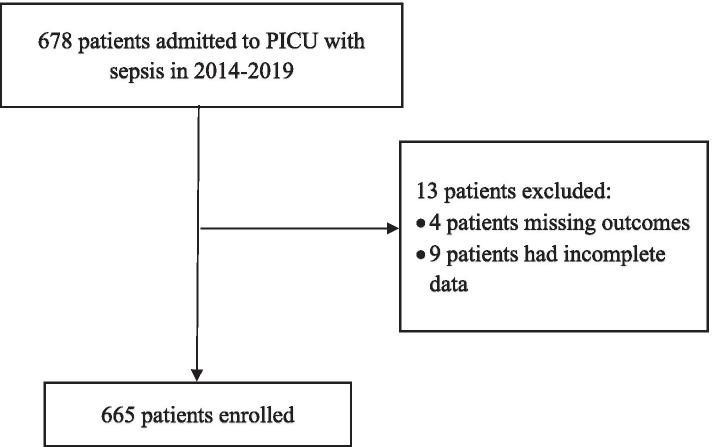
Study Population Flowchart

We retrospectively collected patients’ data from 1st January 2014 to 31st December 2019 who had been diagnosed with sepsis and admitted to the PICU in our tertiary hospital (Dr. Sardjito Hospital, Yogyakarta, Indonesia). We defined sepsis using the Goldstein 2005 criteria which stated sepsis as SIRS followed by infection. All patients were screened during admission at the PICU for sepsis using the Goldstein 2015 definition. The definition of SIRS included a body temperature of > 38.5 °C or < 36.0 °C, tachycardia, mean respiratory rate > 2 SD above normal for age, and a leukocyte count elevated or depressed for age or >  10% immature neutrophils [2]. All PICU sepsis management algorithms were based on Indonesian Pediatric Society guidelines which referred to ACCM/PALS haemodynamic support guidelines. Included patients were those who were admitted to the PICU with sepsis as one of the diagnoses or who were diagnosed with sepsis later during their stay in the PICU. We assumed that all patients who were diagnosed with sepsis later during their stay in PICU had hospital acquired sepsis. All patients were followed up until they reached death as an outcome or date of PICU discharge. Patients with missing medical records or incomplete data related with sepsis criteria and evaluated factors were excluded from the analysis (Table 1). From 678 patients admitted to PICU with sepsis in 2014–2019, 665 patients were enrolled to the study and 13 patients were excluded. The data lock was done on December 31, 2019 (Fig. 1).

**Table 1:** Characteristics of patients with incomplete medical record or missing data

Characteristics	Patient 1	Patient 2	Patient 3	Patient 4	Patient 5	Patient 6	Patient 7	Patient 8	Patient 9	Patient 10	Patient 11	Patient 12	Patient 13
Sex	Female	Male	Female	Female	Male	Male	Female	Male	Female	Male	Female	Male	Male
Age (years)	9.4	3.6	0.2	5.5	2.0	12.7	1.3	16.3	14.8	1.4	1.8	5.6	4.7
Length of Stay (hours)	NA	36	NA	NA	36	0.5	0.3	264	816	288	96	19	22
Nutritional Status^a^	NA	NA	NA	NA	Under nourished	Overweight	NA	NA	NA	NA	NA	NA	NA
Presence of one or more comorbidity	NA	NA	NA	NA	NA	No	No	No	Yes	No	No	No	Yes
Cerebral Palsy	NA	NA	NA	NA	NA	No	No	No	No	No	No	No	No
Heart Disease	NA	NA	NA	NA	No	No	No	No	No	No	No	No	Yes
Kidney Disease	NA	NA	NA	NA	No	No	No	No	No	No	No	No	No
Autoimmune	NA	NA	NA	NA	NA	No	No	No	No	No	No	No	No
Malignancy	NA	NA	NA	NA	NA	No	No	No	No	No	No	No	No
Congenital Anomaly	NA	NA	NA	NA	NA	No	No	No	Yes	No	No	No	No
Referral cases^b^	No	Yes	NA	Yes	Yes	Yes	Yes	No	No	No	No	No	Yes
Mechanical ventilation	NA	NA	NA	NA	No	No	Yes	Yes	Yes	No	Yes	Yes	Yes
Ventilator duration (days)	NA	NA	NA	NA	0.00	0.00	0.01	0.2	0.8	0.00	0.2	0.8	0.9
Vasoactive drugs	NA	NA	NA	NA	NA	Yes	Yes	Yes	No	Yes	No	Yes	Yes
Septic shock	NA	NA	NA	NA	Yes	Yes	Yes	Yes	No	Yes	NA	No	No
Bacteraemia	NA	NA	NA	NA	NA	Negative blood culture	NA	NA	Positive blood culture	NA	NA	NA	NA
Post-operative patient	NA	NA	NA	NA	NA	NA	NA	NA	No	No	Yes	No	
Fluid Overload Percentage	NA	NA	NA	NA	NA	NA	NA	NA	NA	NA	NA	NA	NA
PELOD Score	NA	NA	NA	NA	6	26	10	1	5	5	6	8	8
Outcome	Decease	Survive	Decease	Survive	Survive	Decease	Decease	Decease	Survive	Survive	Survive	Decease	Decease

^a^Nutritional status was categorized based on WHO weight-for-height growth chart for < 5 years old and BMI-for-age growth age for ≥5 years old

^b^ Referral case was defined as cases came from outer hospital

*PELOD* Pediatric Logistic Organ Dysfunction, *NA* Not Available

### Data collection

We recorded demographic and clinical data of all patients during their admission in the PICU. We searched medical record data since 1st January 2014 to 31st December 2019 in our Central Medical Record Unit using keywords: sepsis diagnosis and pediatric patients. We used keywords through electronic medical records database which only include the diagnosis and age category of the patient to make it easier to search for manual medical record numbers. Then we recorded and opened every medical record to collect the data. The demographic data that we evaluated were age and gender, while the clinical data included nutritional status, morbidity, referred from other hospital (yes/no), post-operative patients (yes/no), the need of mechanical ventilation or vasoactive drugs (yes/no), bacteremia, and occurrence of fluid overload.

Patient’s nutritional status was classified according to the World Health Organization (WHO) growth charts: Weight-for-Height curve for children younger than 5 years old or body mass index (BMI)-for-age curve for children 5 years old or older. Children were categorized into having good nutritional status (− 2 ≤ z < 2 SD), being under-nourished (z < − 2 SD) or overweight (z > 2 SD) [9]. Post-operative patients were patients that had undergone surgery before admitted to the PICU. Comorbidity was defined as any chronic condition that has been diagnosed and treated before the patient was diagnosed with sepsis, such as congenital and acquired heart disease, cerebral palsy, malignancy, kidney disease, autoimmune disease and congenital anomaly. All patient’s comorbidties including cerebral palsy in this study were previously diagnosed by an expert in our setting and have had routinely treatment in our pediatric outpatient and also underwent comprehensive treatment with other departments before admission to the PICU.

For children who needed invasive mechanical ventilation or advanced circulatory supports, administrations of both interventions were recorded. The ventilator duration was defined as the total duration of ventilator use during PICU admission (in days). Patients were classified as having septic shock if hemodynamic instability occurred, as indicated by one or more of the following signs: a decrease or change in consciousness, prolonged capillary filling time (> 2 s), decreased strength of peripheral pulses, cold extremities, or bounding pulses, wide pulse pressure differences, or decreased urinary output (< 1 ml/kg/hour), or hypotension [4, 6, 7]. We defined bacteremia as the positive growth of blood culture taken at the first time sepsis diagnosis was established after confirmation that the bacterial isolate was the true pathogen. We performed blood culture testing when the patient met the criteria of sepsis or the doctor in charge had been declared that the patient having sepsis. Standard culture techniques were used, which had been validated using BACTEC® 9120 (BD Diagnostics, Sparks, MD, USA). Clinical Pathology standard procedures were followed for bacterial isolation and antibiotic susceptibility testing [10]. To determine whether a positive culture result was a contaminant, the type of isolated organism, time to positivity, and number of positive culture sites were taken into consideration. We assessed the Pediatric Logistic Organ Dysfunction (PELOD) score using PELOD-2 criteria. The PELOD-2 score criteria consist of 5 system organ dysfunction assessments (range 0–30), including neurologic, cardiovascular, renal, respiratory, and hematologic systems [11].

Fluid overload percentage was calculated based on the total fluid input (in milliliter) minus total fluid output (in milliliter) divided by body weight at hospital admission (in kilogram) and multiplied by 100% [12]. Fluid overload percentage was calculated from the first 24 h after PICU admission until discharge or until the seventh day at PICU if the length of stay exceeded 7 days.

### Outcome measurement

For every patient, the follow-up period ended when the outcome, death or PICU discharge was reached. In our PICU, we held routine death conferences to conclude the cause of death of every death case, that was attended by experts and hospital representatives. From the result of it, we concluded that all death cases in this study were due to sepsis. The mortality rate of sepsis was defined as the proportion of patients who died during PICU stay in all children with sepsis who were admitted to the PICU within the study period.

### Data analysis

Continuous data are presented as mean and standard deviation (SD) for normally distributed data or median and quartiles (Q) Q1 and Q3 for skewed data. Categorical variables are presented as counts and percentages. Normality of distributions was checked using Kolmogorov-Smirnoff test. To evaluate which characteristics could be associated with patients’ outcome, Cox proportional hazard regression was performed in pediatric patients admitted to the PICU, with observation time starting at the time of diagnosis of sepsis (whether at admission or later after admission) and ending at the time of outcome occurrence (decease or survive) or the study closing date. If *p* values in the univariable analysis were <  0.05, then the variables were included into a multivariable analysis to estimate the mutually independent associations between selected characteristics and outcomes. Kaplan-Meier survival curves were created separately for all statistically significant characteristics. Data were analyzed using IBM SPSS Statistics 23rd version.

### Ethics approval and inform consent

This study was approved by the Medical and Health Research Ethics Committee of the Faculty of Medicine, Public Health and Nursing, Universitas Gadjah Mada, Yogyakarta, Indonesia KE/FK/ 0860/EC/2019. As a teaching hospital, all patients or guardians are required to sign an informed consent form related to data usage for education and research purposes at patients’ admission time.

## Results

### Patient characteristics

Demographic characteristics of the patients are shown in Table 2. Overall, there were 364 (54.7%) boys and 301 (46.3%) girls. The median age of patients admitted to PICU was 1.8 with interquartile range (IQR) ±8.36 years old and the median length of stay was 144 h (1–1896 h). From 665 patients, more than half 391 patients (58.8%) had good nutritional status, 216 patients (32.4%) were undernourished and only 58 patients (8.8%) were overweight. As many as 277 patients (41.7%) had chronic disease comorbidity, and multiple comorbidities were quite common. The most common comorbidities were heart diseases (105 patients) and kidney diseases (65 patients). Other reported comorbidities were congenital anomalies in 64 patients (9.6%), malignancy in 52 patients (7.8%), cerebral palsy in 36 patients (5.4%) and autoimmune disorders in 15 patients (2.3%). As many as 486 patients (73.1%) were referred from other primary or secondary care hospitals.

**Table 2:** Characteristics of research subjects

Characteristics	Total *N* = 665 (%)	Outcome				*p* value
		Deceased		Survived		
		n	%	n	%	
Sex						
Male	364 (54.7)	208	54.0	156	55.7	0.666
Female	301 (46.3)	177	46.0	124	44.3	
Age, median ± IQR (years)	1.80 ± 8.36	1.80 ± 9.00		1.90 ± 7.28		0.599
Length of Stay, median ± IQR (hours)	144.00 ± 216.00	72.00 ± 193.00		192.00 ± 192.00		< 0.001
Nutritional Status^a^						
Good nutritional status	391 (58.8)	223	57.9	168	60.0	0.680
Under nourished	216 (32.4)	129	33.5	87	31.1	
Overweight	58 (8.8)	43	8.6	25	8.9	
Presence of one or more comorbidity						
Yes	277 (41.7)	184	47.8	93	33.2	< 0.001
No	388 (58.3)	201	52.2	187	66.8	
Cerebral Palsy	36 (5.4)	10	2.6	26	9.3	< 0.001
Heart Disease	105 (15.8)	77	20	28	10	< 0.001
Kidney Disease	65 (9.8)	47	12.2	18	6.4	0.013
Autoimmune	15 (2.3)	11	2.9	4	1.4	0.221
Malignancy	52 (7.8)	37	9.6	15	5.4	0.044
Congenital Anomaly	64 (9.6)	50	13.0	14	5.0	< 0.001
Referral cases^b^						
Yes	486 (73.1)	272	70.6	214	76.4	0.097
No	179 (26.9)	113	29.4	66	23.6	
Mechanical ventilation						
Yes	570 (85.7)	371	96.4	199	71.1	< 0.001
No	95 (14.3)	14	3.6	81	28.9	
Ventilator duration. median ± IQR (days)	4.00 ± 3.00	3.00 ± 7.00		2.00 ± 5.79		0.394
Vasoactive drugs						
Yes	450 (67.7)	311	80.8	139	49.6	< 0.001
No	205 (33.3)	74	19.2	141	50.4	
Septic shock						
Yes	490 (73.7)	318	82.6	172	61.4	< 0.001
No	175 (26.3)	67	17.4	108	38.6	
Bacteremia						
Positive blood culture	124 (18.6)	67	17.4	57	20.4	0.001
Negative blood culture	313 (47.1)	163	42.3	150	53.6	
No data	228 (34.3)	155	40.3	73	26.1	
Post-operative patient						
Yes	147 (22.1)	61	15.8	86	30.7	< 0.001
No	518 (77.9)	324	84.2	194	69.3	
Fluid Overload Percentage						
≤ 10%	472 (71.0)	202	52.5	270	96.4	< 0.001
> 10%	193 (29.0)	183	47.5	10	3.6	
PELOD Score	7.00 ± 5.00	8.00 ± 5.00		5.00 ± 5.00		< 0.001

^a^Nutritional status was categorized based on WHO weight-for-height growth chart for < 5 years old and BMI-for-age growth age for ≥5 years old

^b^ Referral case was defined as cases from other hospitals

*IQR* Interquartile Range, *PELOD* Pediatric Logistic Organ Dysfunction

Some interventions were commonly performed in our patients. About 570 patients (85.7%) who were admitted to the PICU needed mechanical ventilation support, with median duration of 4.00 days (0.00–65.00 days). About two-thirds of all patients (67.7%) needed vasoactive drugs during the treatment and 147 patients (22.1%) were post-operative patients. As many as 490 patients (73.7%) progressed to septic shock. From all patients, 29% had fluid overload percentage >  10%.

A total of 134 patients (20.2%) in our study had a positive blood culture. The most common organism found was Staphylococcus sp. The duration of stay in the PICU ranged from 1 to 1896 h with median time of 264 h. Three hundred and eighty five patients (57.9%) died during this study period. We found that the presence of chronic disease comorbidities, the need of invasive mechanical ventilation support and vasoactive drugs, septic shock, post-operative patient and fluid overload percentage > 10% to be significantly different between those that died and those that survived.

### Survival of sepsis patients

**Fig. 2:**
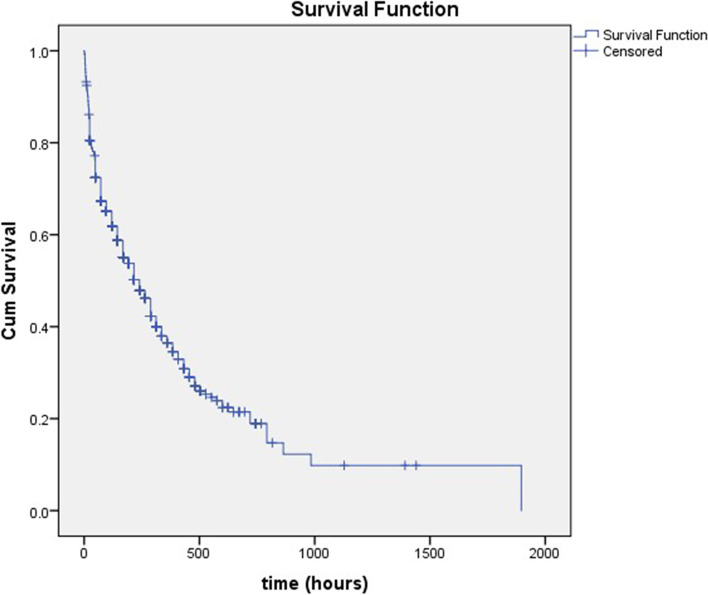
Overall Survival Graphic

Overall, the survival rates of patients in our study at 12 h, 24 h and 48 h were 91.6, 80.6 and 72.4%. Between 48 h (2 days) until 216 h (9 days) of admission, the survival rate was above 50%. Meanwhile, at 240 h (10 days) of admission, the survival rate became 47.8% and it worsened with each additional day. The length of stay of all patients in this study was 433 ± 45 h (18 days) (Fig. 2).

### Prognostic factors

Table 3 shows the demographic and clinical characteristics with survival of PICU patients. Multivariable Cox proportional hazards modeling showed that mortality was associated with the following factors: presence of congenital anomaly (HR 1.4, 95% CI: 1.0–1.9), fluid overload percentage (HR 9.6, 95% CI: 7.4–12.6), the need of invasive mechanical ventilation support (HR 2.7, 95% CI 1.6–4.6), vasoactive drugs (HR 1.5, 95% CI 1.2–2.0) and PELOD score (HR 1.13, 95% CI: 1.10–1.16 and 1.06 95% CI: 1.03–1.09 adjusted). On the contrary, we found that cerebral palsy was associated with lower mortality (HR 0.3, 95% CI: 0.1–0.5). Similarly, post-operative patients showed lower mortality (HR 0.4, 95% CI: 0.3–0.6).

**Table 3:** Association of demographic and clinical characteristics with survival of PICU patients

Variables	Crude hazard ratio (95% CI)	***p***-value	Adjusted hazard ratio (95% CI)	***p***-value
Sex (male/female)	1.0 (0.8–1.2)	0.959		
Age (months)	1.01 (0.99–1.03)	0.447		
Nutritional status				
Good nutritional status^a^				
Under nourished	1.0 (0.8–1.3)	0.699		
Overweight	1.2 (0.9–1.8)	0.256		
Referral case	0.9 (0.7–1.1)	0.261		
Chronic Disease Comorbidity	1.3 (1.0–1.5)	0.023	1.3 (1.0–1.7)	0.099
Cerebral Palsy	0.4 (0.2–0.8)	0.005	0.2 (0.1–0.4)	< 0.001
Heart disease	1.4 (1.0–1.7)	0.019	0.8 (0.6–1.1)	0.125
Kidney Disease	1.4 (1.0–1.9)	0.041	0.9 (0.6–1.3)	0.677
Autoimmune	1.3 (0.7–2.3)	0.425		
Malignancy	1.2 (0.8–1.6)	0.399		
Congenital Anomaly	1.5 (1.1–2.0)	0.008	1.4 (1.0–1.9)	0.032
Mechanical ventilation	4.0 (2.3–6.7)	< 0.001	2.7 (1.6–4.6)	< 0.001
Vasoactive drugs	2.7 (2.1–3.4)	< 0.001	1.5 (1.2–2.0)	0.002
Septic shock	2.3 (1.7–3.0)	< 0.001	1.0 (0.6–1.6)	0.924
Post-operative patient	0.3 (0.2–0.4)	< 0.001	0.4 (0.3–0.6)	< 0.001
Fluid Overload Percentage (> 10%)	11.7 (9.1–15.0)	< 0.001	9.7 (7.4–12.6)	< 0.001
PELOD Score	1.13 (1.10–1.16)	< 0.001	1.06 (1.03–1.09)	< 0.001

Univariable and multivariable Cox Proportional Hazards Models; *CI* confidence interval, *PELOD* Pediatric Logistic Organ Dysfunction; ^a^ Reference category

Patients with a congenital anomaly had a higher mortality risk than patients without such comorbidity (HR 1.4, 95% CI: 1.0–1.9). Survival analysis of sepsis patients with fluid overload percentage > 10% showed significant difference in survival rate. Patients with fluid overload percentage > 10% had lower survivals (HR 9.7, 95% CI: 7.4–12.6, *p* <  0.001), and the survival time was significantly shorter (47 ± 4 h).

We found that the survival of patients who needed mechanical ventilation support or vasoactive drug to be significantly lower as compared to those without (HR 2.7, 95% CI 1.6–4.6, p <  0.001) for mechanical ventilation and (HR 1.5 95% CI: 1.2–2.0, p <  0.001) for vasoactive drug use.

## Discussion

We found that in children with sepsis, fluid overload percentage of more than 10%, the need for mechanical ventilation, and the need of vasoactive agent were associated with higher mortality risk. In contrast, children who had cerebral palsy and those who were post-operative patients appeared to have lower risks of death.

Our study included a large number of subjects, with as many as 364 boys and 301 girls in the study. The study was located in one of the main university-based referral hospitals in Indonesia, equipped with a tertiary-care PICU facility, where particularly sepsis patients are referred to. We receive pediatric patients from primary and secondary hospitals in our region. All PICU management algorithms were based on Indonesian Pediatric Society guidelines. This is among the first studies to investigate the association between demographic and clinical characteristics with mortality of children with sepsis who need PICU admission in Indonesia.

There were some aspects to take into consideration about our study. We still use manual medical records. Although 13 patient records had to be excluded, we do not think that this had a major influence on our findings. We tried to minimize all data collection error by adding a second data collector who validated data input process. Our study did not include other factors that might affect the mortality of patients with sepsis, such as time lag to PICU transfer from other hospitals, vasoactive inotropic score, previous treatments, and duration of stay in the previous hospital since mainly our patients were referral cases and there were no data about those factors in our medical records, becoming weakness of our study. In addition, 228 patients did not perform blood culture testing. The patient’s parents/guardian refused the management due to financial problems and limited coverage of health insurance. These are common problems in low-resource country setting. In further generalization of our findings it should be taken into consideration that this was a single-center study and might not reflect the overall situation of pediatric sepsis patients in other low-resource settings.

We showed that fluid overload of > 10% was an independent predictor of pediatric sepsis mortality. This result was similar to a previous study with a study setting in a middle-resource country which showed that fluid overload percentage of > 10.1% in the first 96 h of sepsis patient care was associated with higher mortality in the first 28 days of care [13]. Moreover, this condition can be explained by the integration of the renal hemodynamic and physiological systems. The septic shock mechanism characterized by vasodilation and capillary leak often causes hypoperfusion of organs and tissues. Appropriate fluid administration is predicted to restore vascular tone and peripheral circulation. Remarkably, septic patients who received a restrictive fluid therapy strategy after day 3 had better survival compared to those who did not [5]. Fluid overload in patients with sepsis can also be caused by excessive fluid management, particularlywhen there are no strong indications for such strategy. Rapid fluid administration increases the final diastolic pressure and stimulates the cardiac muscle’s natriuretic peptide release. Natriuretic peptide causes proteoglycans and glycoprotein decay from glycocalyx, thus aggravating glycocalyx damage [14].

We also looked for any association between the need of invasive mechanical ventilation support and mortality. Previous study had identified the relationship between mechanical ventilation and mortality [12]. This current study shows a similar result that there is an association between mechanical ventilation and sepsis patients’ mortality. The need of vasoactive drugs was also a predictive factor for mortality in our subjects. A previous study with smaller sample size reported a significant correlation between vasoactive drugs and mortality (OR 12.24; 95% CI: 4.4–35.4; *p* <  0.001) [15]. It might reflect those who receive inotropic agent were patients with severe condition.

Furthermore, there was an association between congenital anomaly and mortality in patients with sepsis. This finding is consistent with the previous study that revealed an association between patients with congenital anomaly and mortality [16]. It was proposed that sepsis recruited inflammatory mediators that can cause multi organ dysfunction. One major issue during sepsis and septic shock is severely impaired gastrointestinal function, especially gastrointestinal motility, clinically recognized as the paralytic ileus. As a result, impaired gastrointestinal motility is one of the reasons why infections arise during sepsis; however, the exact mechanism of this remains unclear [17].

Moreover, we found an association between PELOD-2 score and mortality in pediatric patients with sepsis. A previous study showed a similar result and had a strong association (p <  0.001) with 74.5% sensitivity, 42.7% specificity, 96.5% NPV, and 7.4% PPV for predicting mortality [18]. A study found that PELOD-2 scores were significantly higher in non-survivors compared to survivors [mean 14.9 (SD 6.1) vs. mean 4.2 (SD 3.2), respectively, *p* <  0.0001] [11].

We found significant association between cerebral palsy comorbidity and survival. It was different from previous studies that found 49% of death in patients with cerebral palsy was attributed to infection [19]. These conditions might be correlated with the severity category according to functional disability in cerebral palsy patients. A previous study showed that patients with cerebral palsy with good nutritional status had a better outcome if they had an infection episode compared to those who did not have good nutritional status [20]. All cerebral palsy patients included in this study had good nutritional status because they already underwent comprehensive management in our neurology department. These factors might be the reason for the patient had better survival in this study. Patients with cerebral palsy had lower mortality may be related to they are commonly administered with intracranial dose of antibiotics when they are suspected having sign of infection. This is also the case with post-operative patients. The surgeon tended to immediately give antibiotics after surgery. Patients with a better category in cognition, mobility, manual dexterity, and hearing will have a higher survival after 2 years of age. In this study we did not record the severity of our cerebral palsy patients [5].

We also found an association between surgical intervention and lower mortality rate in patients with sepsis. A previous study found different results in adult patients, where post-operative sepsis was an independent factor of the incidence of mortality at 30-days, 60-days, and 1-year with HR 2.75, 95% CI: 2.14–3.53; HR 2.45, 95% CI: 1.94–3.10; and HR 1.71, 95% CI: 1.46–2.00, respectively [21]. Remarkably, in another study, 31% of those with post-operative sepsis required renal replacement therapy, and 37% required prolonged mechanical ventilation [16]. These differences could be due to differences in the patients included in our study.

Patients in the PICU are susceptible to fluid overload following intravenous fluid administration or deterioration during critical illness. Sepsis and multiple organ failure increase the risk of fluid overload-related complications due to glycocalyx breakdown and pre-existing organ dysfunction. Increased capillary permeability and changes in oncotic gradient pressure are pitfalls in sepsis management that lead to fluid overload [22, 23]. Therefore, fluid administration to patients with sepsis either for resuscitation or maintenance necessitates careful attention by the physician [12].

## Conclusion

PICU mortality is affected by fluid overload percentage of > 10%, the need for mechanical ventilation support, the need of vasoactive drugs, and the presence of congenital anomaly. In septic patients in PICU, those with cerebral palsy and admitted for post-operative care had better survival.

## Data Availability

All data generated or analyzed during this study are included in the submission. The raw data are available from the corresponding author on reasonable request.

## Abbreviations

PICU: Pediatric intensive care unit
PELOD: Pediatric Logistic Organ Dysfunction
HR: Hazard ratio
